# Tooth brushing with fluoridated toothpaste and associated factors among Chinese adolescents: a nationwide cross-sectional study

**DOI:** 10.1186/s12903-023-03506-w

**Published:** 2023-10-18

**Authors:** Zhiying Cui, Wenhui Wang, Yan Si, Xing Wang, Xiping Feng, Baojun Tai, Deyu Hu, Huancai Lin, Bo Wang, Chunxiao Wang, Shuguo Zheng, Xuenan Liu, Wensheng Rong, Weijian Wang

**Affiliations:** 1 Department of Preventive Dentistry, Peking University School and Hospital of Stomatology & National Center for Stomatology & National Clinical Research Center for Oral Diseases & National Engineering Research Center of Oral Biomaterials and Digital Medical Devices, Beijing, China; 2 Chinese Stomatological Association, Beijing, China; 3 Shanghai Ninth People’s Hospital, Shanghai Jiao Tong University School of Medicine, Shanghai, China; 4 School & Hospital of Stomatology, Wuhan University, Wuhan, China; 5 West China School of Stomatology, Sichuan University, Chengdu, China; 6 Guanghua School of Stomatology, Hospital of Stomatology, Sun Yat-sen University, Guangzhou, China; 7 Chinese Center for Disease Control and Prevention, Beijing, China

**Keywords:** Tooth brushing, Fluoridated toothpaste, Adolescent, Oral hygiene habit

## Abstract

**Background:**

Tooth brushing with fluoridated toothpaste has become the most important way to provide the anti-caries effect of fluoride around the world. China has promoted the use of fluoridated toothpaste since 1989. However, there are few studies on the national profile of use of fluoridated toothpaste and related factors in Chinese adolescents. We carried out this study to investigate oral hygiene behaviours, especially the status of tooth brushing with fluoridated toothpaste and its correlates among adolescents, based on data from the latest Nation Oral Health Survey in mainland China.

**Methods:**

This cross-sectional study recruited 118,601 participants aged 12–15 years using multistage stratified sampling. Questionnaires were completed by students at school. Data employed in analyses were extracted from the questionnaire, including information on tooth brushing, fluoridated toothpaste, dental floss, sociodemographic factors, fluoride knowledge and attitude towards regular dental check-ups. A binary logistic regression was performed to compute the odds ratios (OR). Tooth brushing twice daily with fluoridated toothpaste was the dependent variable. Sociodemographic factors, fluoride knowledge, attitude towards regular dental check-ups, dental visit experience and perceived oral health were the independent variables. *P* < 0.05 was considered statistically significant.

**Results:**

A total of 32.6% of participants brushed their teeth twice daily, 7.4% used fluoridated toothpaste, and 3.9% cleaned their teeth twice daily with fluoridated toothpaste. The logistic regression showed the probability of twice-a-day tooth brushing with fluoridated toothpaste was higher among these groups: females (OR: 1.141; 95%CI: 1.072–1.214), 15-year-olds (OR: 1.786; 95%CI: 1.634–1.952), from urban areas (OR: 1.389; 95%CI: 1.288–1.497), without siblings (OR: 1.351; 95%CI: 1.259–1.450), with an educated father (OR: 1. 605; 95%CI: 1.442–1.788) and mother (OR: 1.706; 95%CI: 1.530–1.903), having dental visit experiences (OR: 1.702; 95%CI: 1.589–1.823), rating one’s oral health as good (OR: 2.341; 95%CI: 2.083–2.631), having fluoride knowledge (OR: 4.345; 95%CI: 4.034–4.678) and having a positive attitude towards regular dental check-ups (OR: 1.589; 95%CI: 1.460–1.729).

**Conclusions:**

The oral hygiene behaviours of Chinese adolescents were undesirable. Twice daily tooth brushing with fluoridated toothpaste was significantly associated with sociodemographic factors, fluoride knowledge, and attitudes towards regular dental check-ups.

**Supplementary Information:**

The online version contains supplementary material available at 10.1186/s12903-023-03506-w.

## Background

In the past 50 years, the widespread application of fluoride has significantly reduced the incidence of dental caries, fundamentally improved people’s oral health, and enhanced the general health and quality of life of people worldwide. Brushing teeth with fluoridated toothpaste has become the most important way to provide the anti-caries effect of fluoride around the world [1–3]. The World Health Organization Global Dental Programme advocates the effective use of fluoride as the basic method of caries prevention in the 21st century and emphasizes the importance of using affordable fluoridated toothpaste [4]. It was found that fluoridated toothpaste reduces DMFS in children and adolescents by 26-28% within 3 years [5]. In addition, many systematic analyses have confirmed that the use of fluoridated toothpaste can significantly reduce the risk of dental caries in children and adolescents [6–10].

It is worth noting that the beneficial effect of fluoridated toothpaste is related to the frequency of brushing teeth at least twice a day [1]. In 2001, the Centers for Disease Control and Prevention (CDC) of the United States proposed that brushing twice a day with fluoridated toothpaste is the basic recommendation to prevent dental caries [11]. Additionally, the European Academy of Paediatric Dentistry (EAPD) recommends twice-daily brushing teeth with fluoridated toothpaste as the basic measure to prevent dental caries, regardless of the age of the adolescent and the risk of caries [12].

China has promoted the use of fluoridated toothpaste since 1989, but the overall application of fluoridated toothpaste has been relatively low because of large population needs, implementation barriers, socio-environmental factors and limited capacity of the oral health system in China [13]. Scholars have found that factors such as gender, family economic status, parental education or occupation, and oral health knowledge and attitude, and oral health assessments are associated with behaviour of brushing teeth with fluoridated toothpaste twice a day [14, 15]. However, there are few studies on the national profile of brushing teeth with fluoridated toothpaste twice a day and related factors in Chinese adolescents [16]. Therefore, it is necessary to obtain such information as a basis from which to launch oral health promotion programs among Chinese adolescents.

The 4th National Oral Health Survey (NOHS) is the latest cross-sectional survey supported by the Scientific Research Fund of the National Health Commission of the People’s Republic of China and organized by the Chinese Stomatological Association (CSA), with cooperation from the Chinese Center for Disease Control and Prevention [17]. This article aims to investigate the oral hygiene behaviours and explore the related factors to tooth brushing with fluoridated toothpaste in adolescents aged 12 to 15 years old by using data from the 4th NOHS. Our hypothesis was that twice daily tooth brushing with fluoridated toothpaste among Chinese adolescents was associated with demographic characteristics, socioeconomic factors, dental knowledge and attitude, dental visit experience and self-assessment of oral health.

## Materials and methods

### Sampling and sample size

This cross-sectional study analysed data from the 4th NOHS, which covered all 31 provinces, municipalities and autonomous regions of mainland China. Data collection for the survey was carried out in 2015 and 2016. Regarding the findings of the 3rd NOHS in 2005, the prevalence of dental caries experience among children aged 12 years was 28.9%. The 95% confidence interval (CI) was set at 15% with two sides, and the design effect was set at 4.5 which was based on data from previous national oral health surveys [17]. To account for the stratification factor and an anticipated response rate of 80%, the sample sizes were 28,365 each for the 12-, 13-, 14-, and 15-year age groups. Participants were selected using multistage stratified cluster sampling. First, each province was divided into two strata, urban and rural areas, and then two districts or counties were randomly selected from each stratum. Second, from the list of secondary schools provided by the local government, three secondary schools were randomly selected in each district or county. Third, in each secondary school, 320 children aged 12, 13, 14, and 15 with a male-to-female ratio of 1:1 were selected using cluster sampling, with 80 children in each age group. A target sample of 3840 participants was initially set for each province, for a total of 119,040 children nationwide. The protocols for the 4th NOHS were reported in the Chinese Journal of Dental Research, the official journal of the CSA [17].

### Questionnaire

Questionnaires were completed by students at school. Trained investigators explained the questionnaires to the students prior to completion. The questionnaire was designed to obtain information about sociodemographic factors, dietary habits, oral hygiene practice, smoking, dental visit experience, oral health knowledge and attitude, self-assessment of oral health and general health, and quality of life. Data employed in this study were extracted from the questionnaire, including information on the frequency of tooth brushing, use of fluoridated toothpaste and dental floss, sociodemographic factors (gender, age, registered residence, number of children in the family, paternal and maternal education), self-assessment of oral health, fluoride knowledge and attitude towards regular dental check-ups. Concerning knowledge of fluoride, one question was asked: “fluoride is not effective for protecting teeth”, participants with the response “not correct” were considered to have knowledge of fluoride. Regarding oral health attitude, one question “regular dental check-ups are essential” was used. Participants who agreed the statement “regular dental check-ups are essential” had a positive attitude, and those who disagreed, didn’t matter or didn’t know were considered to have negative attitude (Additional file 1).

### Statistical analysis

Statistical Package for Social Sciences (SPSS), version 26 (IBM Corp., Armonk, NY) was used for data processing and statistical analysis. First, descriptive analysis was performed for the full study sample to show the number and percentage of adolescents with different oral hygiene behaviours. Then, chi-square tests were performed to test if statistically significant relationships existed between twice-a-day tooth brushing with fluoridated toothpaste and related variables. Variables with p < 0.1 in the chi-square test were included in the multivariable analysis. Furthermore, a binary logistic regression was performed to examine the association between the dependent variable and the independent variables. Whether participants brushed their teeth twice-a-day with fluoridated toothpaste was the dependent variable. Sociodemographic factors, fluoride knowledge, attitude towards regular dental check-ups, dental visit experience and perceived oral health were the independent variables.

### Ethics approval and consent to participate

Ethical approval (Approval No: 2014-003) for the study was received from the Ethics Committee of the Chinese Stomatological Association and written informed consent was obtained from parents or legal guardians of each participant. All methods were carried out in accordance with relevant guidelines and regulations of the Ethics approval under Declarations of Helsinki.

## Results

### Descriptive characteristics

A total of 118,601 students completed the questionnaires. The response rate is 99.6%. Of all participants, 50% were female; the distributions in the 12-, 13-, 14- and 15-year-old groups were 23.5%, 26.1%, 25.9%, and 24.6%, respectively; and 40.6% were from urban areas (Table 1). Excluding the effect of missing values in some independent variables related to this study, a total of 118,565 adolescents were included in the final multivariable analysis, representing approximately 100% of the original sample. The independent variables with missing data are shown in Table 2.

**Table 1:** Distribution of oral hygiene behaviours according to gender, age, and registered residence

			Frequency of toothbrushing (%)			Use of fluoridated toothpaste (%)				Frequency of flossing (%)5			
		** N (percentage)**	**≥ twice/day**	**once/day**	**Not everyday**	**Yes**	**No**	**Don’t know**	**No response**	**Daily**	**Every week**	**Occasionally**	**Never**
**Gender**	Male	59,263 (50.0)	26.4	54.0	19.6	8.1	5.8	71.7	14.4	0.7	0.8	9.0	89.5
	Female	59,338 (50.0)	38.7	53.7	7.6	6.8	5.0	82.8	5.3	0.4	0.5	8.0	91.1
			*P < 0.001*			*P < 0.001*				*P < 0.001*			
**Age**	12 years old	27,821 (23.5)	31.9	50.9	17.2	5.9	4.8	76.3	12.9	0.6	0.7	8.3	90.4
	13 years old	30,961 (26.1)	31.7	53.3	15.0	6.0	5.0	78.0	10.9	0.6	0.7	9.0	89.7
	14 years old	30,691 (25.9)	32.6	54.8	12.5	7.6	5.5	78.1	8.8	0.6	0.7	8.4	90.3
	15 years old	29,128 (24.6)	34.1	56.2	9.7	10.3	6.3	76.5	7.0	0.5	0.5	8.1	90.9
			*P < 0.001*			*P < 0.001*				*P < 0.001*			
**Registered residence**	Rural	70,491 (59.4)	23.9	59.7	16.3	5.5	4.6	77.8	12.0	0.3	0.4	5.3	94.0
	Urban	48,103 (40.6)	45.3	45.2	9.5	10.2	6.6	76.5	6.7	0.9	1.0	13.2	84.9
			*P < 0.001*			*P < 0.001*				*P < 0.001*			

**Table 2:** Distribution and associations of twice-a-day tooth brushing with fluoridated toothpaste under different variables. (*N* = 118,601)

	Chi-square tests			Binary logistic regression	
	Toothbrushing twice daily with fluoridated toothpaste (%)		*P* value	OR (95% CI)	*P* value
	Yes	No			
**Gender**	**3.9**	96.1	0.052		
Male	3.8	96.2		1	
Female	4.0	96.0		1.141 (1.072–1.214)	< 0.001
**Age**			< 0.001		
12 years old	3.0	97.0		1	
13 years old	3.1	96.9		1.034 (0.938–1.139)	**0.504**
14 years old	3.9	96.1		1.329 (1.211–1.459)	< 0.001
15 years old	5.5	94.5		1.786 (1.634–1.952)	< 0.001
**Registered residence**			< 0.001		
Rural	2.2	97.8		1	
Urban	6.3	93.7		1.389 (1.288–1.497)	< 0.001
**Number of children in the family**			< 0.001		
>1	2.4	97.6		1	
1	6.4	93.6		1.351 (1.259–1.450)	< 0.001
**Paternal education level**			< 0.001		
Junior high school and below	2.3	97.7		1	
Senior high school	5.4	94.6		1.340 (1.227–1.462)	< 0.001
College and above	10.8	89.2		1.605 (1.442–1.788)	< 0.001
Unaddressed	2.4	97.6		1.014 (0.865–1.19)	**0.860**
**Maternal education level**			< 0.001		
Junior high school and below	2.5	97.5		1	
Senior high school	6.2	93.8		1.384 (1.267–1.512)	< 0.001
College and above	11.4	88.6		1.706 (1.530–1.903)	< 0.001
Unaddressed	2.0	98.0		0.781 (0.662–0.922)	**0.003**
**Dental visit experience**			< 0.001		
No	2.2	97.8		1	
Yes	5.5	94.5		1.702 (1.589–1.823)	< 0.001
**Self-assessment of oral health**			< 0.001		
Very poor or poor	2.1	97.9		1	
General	2.8	97.2		1.315 (1.168–1.481)	< 0.001
Good or very good	6.2	93.8		2.341 (2.083–2.631)	< 0.001
**Fluoride is not effective for protecting teeth**			< 0.001		
Correct or don’t know	1.4	98.6		1	
Not correct	7.6	92.4		4.345 (4.034–4.678)	< 0.001
**Regularly dental check-ups are essential**			< 0.001		
Disagree or don’t know	2.0	98.0		1	
Agree	4.6	95.4		1.589 (1.460–1.729)	< 0.001

*OR* Odds ratio

*CI* Confidence interval

**Fig. 1:**
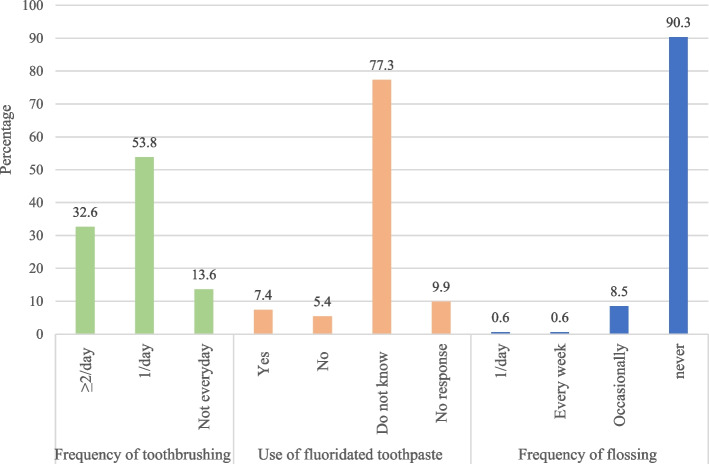
Distribution of oral hygiene behaviours in 12–15 years Chinese schoolchildren

Figure 1 shows the distribution of oral hygiene behaviours in Chinese adolescents. Among the total participants, 32.6% brushed their teeth twice a day, 53.8% brushed once a day, and 13.6% cleaned less than once a day. Additionally, 7.4% of the participant reported using fluoridated toothpaste, 5.4% reported using non-fluoridated toothpaste, and as high as 77.3% did not know whether their toothpaste contained fluoride. The frequency of adolescents using dental floss is very low, with only 0.6% using it every day and more than 90% answering that they never used dental floss.

### Distribution of oral hygiene behaviours according to gender, age, and registered residence

The prevalence of tooth brushing twice daily or more often and using fluoridated toothpaste increased with age (*P* < 0.001; *P* < 0.001). Female students showed the higher prevalence of twice daily tooth brushing (*P* < 0.001) and lower prevalence of using fluoridated toothpaste (*P* < 0.001) and dental floss (*P* < 0.001). Good oral hygiene behaviours were found in students whose registered residence was urban. Details on the distribution of oral hygiene behaviours according to gender, age, and registered residence are listed in Table 1.

### Distribution and associations of twice-a-day tooth brushing with fluoridated toothpaste under different variables

A total of 3.9% of participants cleaned their teeth twice daily with fluoridated toothpaste. In the univariate analysis, all factors were significantly correlated with twice-a-day tooth brushing with fluoridated toothpaste (Table 2). The results of the multivariable analysis showed that the following groups had greater odds of cleaning their teeth twice-a-day with fluoridated toothpaste: females (OR: 1.141; 95% CI: 1.072–1.214), 15-year-olds (OR: 1.786; 95% CI: 1.634–1.952), children whose registered residence being urban areas (OR: 1.389; 95% CI: 1.288-1. 497), without siblings (OR: 1.351; 95% CI: 1.259–1.450), whose father had a higher level of education (OR: 1.340; 95% CI: 1.227–1.462 & OR: 1.605; 95% CI: 1.442–1.788), whose mother had higher education (OR: 1.384, 95% CI: 1.267–1.512 & OR: 1.706; 95% CI: 1.530–1.903), who had dental visit experiences (OR: 1.702; 95% CI: 1.589–1.823), rating one’s oral health as general or good (OR: 1.315; 95% CI: 1.168–1.481 & OR: 2.341; 95% CI: 2.083–2.631), having fluoride knowledge (OR: 4.345; 95% CI: 4.034–4.678), and having a positive attitude towards regular dental check-ups (OR: 1.589; 95% CI: 1.460–1.729) (Table 2).

## Discussion

The findings of this study showed that oral hygiene behaviours among 12–15 years Chinese schoolchildren were not desirable. In the decade from 2005 to 2015, the proportion of 12-year-olds brushing their teeth twice a day increased from 28.4% [18] to 31.9% (Table 1). However, the percentage of 12-year-olds using fluoridated toothpaste decreased from 11.9% [18] to 5.9% (Table 1). Children’s twice daily tooth brushing with fluoridated toothpaste had a significant relationship with socioeconomic factors, fluoride knowledge, and attitudes towards regular dental check-ups after adjusting age, gender and registered residence. This study suggests that interventions are essential to enhance 12–15 years Chinese schoolchildren’s oral hygiene behaviours. To the best of our knowledge, this is the first report on these issues in mainland China. The results provide a strong nationwide basis from which to commence oral health promotion programs.

Tooth brushing is the most effective method of oral hygiene, and the universally recommended frequency has been twice a day [19]. However, less than 1/3 of 12–15 years Chinese schoolchildren brushed their teeth twice a day. Compared to the findings of other studies on similarly aged participants, the finding of this study is higher than that in Iran (13–16 years, 20.1%) [20], but much lower than that in Malaysia (13–17 years, 87%) [21], Indonesia (11–18 years, 89.2%) [22], and the United States (12–15 years, 65%) [23].

Based on strong scientific evidence, cleaning teeth twice a day with fluoridated toothpaste is the most important tool for self-oral care [1, 11, 12]. Only 3.9% of adolescents had such good oral hygiene behaviour in the study. This figure is believed to be lower than the actual rate because as many as 77.3% of adolescents are not sure whether their toothpastes contain fluoride or not. On the other hand, the result of 77.3% indirectly indicates that adolescents do not care about the importance of fluoridated toothpaste or do not pay attention to information regarding fluoridated toothpaste. In Finland, 70% of adolescents knew that fluoride is effective in preventing caries, and 50% brushed their teeth with fluoridated toothpaste at least twice a day [14]. The big difference in twice daily toothbrushing with fluoridated toothpaste between Chinese and Finnish adolescents may be related to the lack of fluoride knowledge, except the difference in sociodemographic characteristics of participants.

What is more noteworthy is that less than 1/10 of adolescents stated using dental floss, and only 0.6% used it every day. Some reports have been published on the use of dental floss in adolescents. The proportion of adolescents who flossed was 33% in Sweden [15], 50.8% in Portugal [24], and 52.3–62.3% in Greece [25], who flossed weekly was 22.3% in Hong Kong [26], and who flossed daily was 16.1% in Nigeria [27].

These large differences in results between this study and other studies may be due to the different study designs, methodologies, sites, times and backgrounds of the samples. However, the prevalence of twice-a-day tooth brushing, usage of fluoridated toothpaste and usage of dental floss among Chinese adolescents is not desirable.

The study showed that tooth brushing twice-a-day with fluoridated toothpaste was more common in girls than boys. Gender differences have been reported by many studies in this field [15, 20, 28–30]. One possible reason is that, compared to male students, female students care more about and pay more attention to their health as an effective factor to increase the beauty and appearance of the body [20, 31]. Furthermore, some authors have explained these gender behaviour differences according to the social and psychological impacts of oral health, finding that women perceived oral health as having a greater impact on their quality of life in general than men did [32].

We found that older children had higher odds of having good oral hygiene practices. Maes et al. reported that the prevalence of twice-a-day tooth brushing seemed to increase with increasing age in many countries [30]. The effect of age may be because as adolescents grow up, they could have more opportunities to obtain oral health knowledge. At the same time, they may attach more importance to oral hygiene as their self-esteem gradually increases [24].

As we expected, adolescents from urban areas had a higher prevalence of good oral hygiene practices. The association between higher socioeconomic status and better oral health, including oral health behaviour, has been investigated in many studies [16, 20, 22, 30]. Generally, people from urban areas have higher socioeconomic status in China; hence, their lifestyle behaviours are healthier due to good financial resources and more access to health care.

After controlling for the possible confounding of demographic variables (gender, age, registered residence), there were still positive relationships between tooth brushing twice a day with fluoridated toothpaste and high parental education, as well as having only one child in the family. These results are similar to those of other studies [20, 30, 33–35]. Highly educated parents may indicate higher health literacy and occupational status. A study collecting data from 32 countries showed a strong association between a high prevalence of more-than-once-a-day tooth brushing with high occupational status and family affluence in all countries studied [30]. On the other hand, educated parents may be considered positive role models for children regarding oral health behaviours. The number of children in a family is an indicator of family economic conditions. Parents may pay more attention and allocate more resources to children’s health in families with only one child. Some researchers reported that tooth decay was more frequently observed among children from families with a higher number of children [36–38].

According to traditional knowledge-attitude-behaviour models (KAB model; or, sometimes KAP model, referring to knowledge, attitude, and practice) on changing behaviour, favourable health behaviour could be associated with good knowledge and attitude [39]. Such positive relationships between oral hygiene practices and knowledge or attitude among adolescents have been reported both in longitudinal and cross-sectional studies [14, 15, 39–41]. Our study also confirmed this issue. The odds of good oral hygiene practices among adolescents with correct fluoride knowledge were more than 4 times that of adolescents without fluoride knowledge, and having fluoride knowledge was the strongest factor.

Perceived oral health was associated with oral hygiene practices. Chinese adolescents who rated their own oral health as good had better oral hygiene behaviours. This is in line with Jensen’s study [15]. Good oral health assessed by children themselves could reflect the real situation of their oral health, and good oral health has been reported to be related to good oral hygiene behaviours in many studies [15, 20, 33]. In the present study, dental visit experience and a positive attitude towards regular dental check-ups were associated with good oral hygiene practices. Those adolescents who believed that regular dental check-ups were essential indicated more opportunities for them to access dental services, which in turn led to more opportunities for dental knowledge. Japanese scholars have found that dental clinics were the most common source of oral hygiene knowledge for Japanese university students [42].

This study also has some limitations. First, this is a cross-sectional study, so we cannot infer causal relationships. Second, our study mainly explored individual factors affecting oral hygiene behaviours, but contextual factors, such as the availability and price of fluoridated toothpaste and geographical area, were not considered. Third, the data used are based on the self-report of adolescents, and it is possible that they could potentially give more socially desirable responses to some questions rather than accurate responses, making reporting bias a possibility. Fourth, weighting was not performed for all analyses, as specific demographic data from 12-to-15-year-olds were not available for the same period.

This study was carried out on a national representative sample of 12- to 15-year-old schoolchildren in mainland China, thereby showing the patterns of oral hygiene behaviours and related beneficial factors for this group. The findings suggest that interventions are urgently needed to enhance oral hygiene behaviours through school-based oral health education programs among Chinese adolescents. Adolescence is an important period for learning, and habits formed during adolescence may continue into adulthood. Regardless, it should be emphasized that twice-a-day tooth brushing with fluoridated toothpaste is essential when designing the content of oral health education and when counselling adolescents.

## Conclusion

The present study found that the rates of twice-a-day tooth brushing, the usage of fluoridated toothpaste, and the usage of dental floss among 12–15 years Chinese schoolchildren are low. Factors that increased the odds of twice-a-day tooth brushing with fluoridated toothpaste are being female, being older, registered residence being urban, being without siblings, having educated parents, having fluoride knowledge, having a positive attitude towards regular dental check-ups, having dental visit experience, and rating one’s own oral health as good. Having fluoride knowledge was the strongest factor to explain twice-a-day tooth brushing with fluoridated toothpaste.

### Supplementary Information

**Additional file 1.**

## Data Availability

The data that support the findings of this study are available from the National Health Commission of the People’s Republic of China but restrictions apply to the availability of these data, which were used under license for the current study, and so are not publicly available. Data are however available from the authors upon reasonable request and with permission of the National Health Commission. Wenhui Wang (wangwenhui@pkuss.bjmu.edu.cn) should be contacted if someone wants to request the data from this study.

## Abbreviations

CDC: Centers for Disease Control and Prevention
CI: confidence interval
CSA: Chinese Stomatological Association
EAPD: European Academy of Paediatric Dentistry
NOHS: National Oral Health Survey
OR: odds ratios
