# Supplementary material for: Tooth brushing with fluoridated toothpaste and associated factors among Chinese adolescents: a nationwide cross-sectional study
Source: BMC Oral Health. 2023 Oct 18;23:765. doi: 10.1186/s12903-023-03506-w (PMC10585760; doi:10.1186/s12903-023-03506-w)
Supplement: Supplementary file 1 — Additional file 1. [file 12903_2023_3506_MOESM1_ESM.docx]

The Fourth National Oral Health Questionnaire Survey (Students) in 2015

Respondent ID Number:

School:

Grade:

Class:

Respondent Name:

Date of Investigation:

Investigator Number:

Dear students,

In order to further improve oral health care for children and adolescents, we would like to know your thoughts and practices regarding oral health care. This survey is unrelated to your academic performance, and your response will not be shared with your parents and teachers. Please answer truthfully according to the question requirements. Thank you!

**Requirements:** Please underline "√" in front of "□" in the corresponding choice in multiple-choice questions.

1. Are you an only child? (choose only one answer)

1) □ Yes 2) □ No

2. What is highest level of education? (choose only one answer)

1) □ Did not go to school 2) □ Primary school 3) □ Junior high school 4) □Senior high school 5) □ Technical secondary school 6) □ Junior college 7) □ Undergraduate 8) □ Master or above 9) □ your father's No father or do not know

3. What is your mother's highest level of education? (choose only one answer)

1) □ Did not go to school 2) □ Primary school 3) □ Junior high school 4) □Senior high school 5) □ Technical secondary school 6) □ Junior college 7) □ Undergraduate 8) □ Master or above 9) □ No mother or do not know

4. Do you brush your teeth? (choose only one answer)

1) □ Yes 2) □ Occasionally or never (respondents who choose 2 should not answer questions 5-7)

5. How many times do you brush your teeth every day? (choose only one answer)

1) □Twice a day or more 2) □ Once a day 3) □ Not every day

6. Do you use toothpaste when you brush your teeth? (Choose only one answer)

1) □ Yes 2) □ No 3) □ Do not know (respondents who choose 2 or 3 should not answer question 7)

7. Do you use fluoridated toothpaste when you brush your teeth? (choose only one answer)

1) □ Yes 2) □ No 3) □ Do not know

8. Do you use floss? (choose only one answer)

1) □ No 2) □ Occasionally 3) □ Weekly 4) □ Daily

9. How often do you consume the following foods or beverages? (only one answer per question)

|  | 6  ≥twice a day | 5  once a day | 4  2-6 times a week | 3  once a week | 2  1-3 times a month | 1  Rarely or never |
| --- | --- | --- | --- | --- | --- | --- |
| 1）Desserts (biscuits, cakes, bread, etc.) and sweets (chocolate, sugary gum, etc.) | □ | □ | □ | □ | □ | □ |
| 2）Sweet beverages (carbonated drinks such as coke, fruit juices such as orange juice and apple juice, non-freshly squeezed juices such as lemonade) | □ | □ | □ | □ | □ | □ |
| 3）Sweetened drinks (milk, yogurt, powdered milk, tea, soy milk, coffee) | □ | □ | □ | □ | □ | □ |

10. Do you smoke? (choose only one answer)

1) □Daily 2) □Weekly 3) □ Rarely or previously smoked 4) □ Never

11. How do you assess your overall physical health? (choose only one answer)

1) □ Very good 2) □ Good 3) □ Average 4) □ Poor 5) □ Very poor

12. How do you assess your oral health? (choose only one answer)

1) □ Very good 2) □ Good 3) □ Average 4) □ Poor 5) □ Very poor

13. Have your teeth been bruised or broken? (choose only one answer)

1) □ Yes 2) □ No 3) □ Do not remember (respondents who choose 2 or 3 should not answer question 14)

14. Where did you hurt your tooth? (Multiple answers can be selected)

1) □ At school 2) □ Outside of school

15. Have you experienced toothache in the past 12 months? (choose only one answer)

1) □ Often 2) □ Occasionally 3) □ Never 4) □ Do not remember

16. Do you have dental visit experiences? (choose only one answer)

1) □ Yes 2) □ Never (respondents who choose 2 should not answer questions 17 and 18)

17. When was your most recent dental visit? (choose only one answer)

1) □ Less than 6 months 2) □ 6 months to 12 months 3) □ More than 12 months

18. What was the primary reason for your most recent dental visit? (choose only one answer)

1) □ Consultation 2) □ Preventive Care 3) □ Treatment 4) □ Do not know

19. Do you think the following statement is true? (choose one answer for each question)

|  | 1  Correct | 2  Incorrect | 8  Do not  know |
| --- | --- | --- | --- |
| 1）Bleeding gums when brushing teeth is normal | □ | □ | □ |
| 2）Bacteria can cause gum inflammation | □ | □ | □ |
| 3) Brushing teeth is not effective in preventing gum inflammation | □ | □ | □ |
| 4）Bacteria can cause dental caries | □ | □ | □ |
| 5）Eating sugary foods can cause dental caries | □ | □ | □ |
| 6）Fluoride is not effective in protecting teeth | □ | □ | □ |
| 7）Pit and fissure sealant can protect teeth | □ | □ | □ |
| 8）Oral diseases may impact overall physical health | □ | □ | □ |

20. What is your opinion on the following statement? (choose one answer for each question)

|  | 1  Agree | 2  Disagree | 3  Does not matter | 4  Do not know |
| --- | --- | --- | --- | --- |
| 1）Oral health is important for one’s life | □ | □ | □ | □ |
| 2）Regular dental check-ups are essential | □ | □ | □ | □ |
| 3）The condition of teeth is innate and not strongly influenced by personal care | □ | □ | □ | □ |
| 4）Preventing dental diseases primarily depends on individual efforts | □ | □ | □ | □ |

21. In the past 6 months, how significant is the impact of oral issues on the following aspects for you? (choose one answer for each question)

|  | 1  Significant impact | 2  Moderate impact | 3  Minor impact | 4  No impact at all | 5  Do not know |
| --- | --- | --- | --- | --- | --- |
| 1) Eating | □ | □ | □ | □ | □ |
| 2) Speaking | □ | □ | □ | □ | □ |
| 3) Brushing your teeth or rinsing your mouth | □ | □ | □ | □ | □ |
| 4) Doing housework | □ | □ | □ | □ | □ |
| 5) Going to school | □ | □ | □ | □ | □ |
| 6) Sleeping | □ | □ | □ | □ | □ |
| 7) Smile with teeth showing | □ | □ | □ | □ | □ |
| 8) Easily upset | □ | □ | □ | □ | □ |
| 9) Socializing | □ | □ | □ | □ | □ |

22. In the last semester, how many times did you have lectures on knowledge of oral health ?

times (please fill in an integer. Fill in N if you do not know or refuse to answer)
